# Advance in Neurotoxicity Research from Development to Aging

**DOI:** 10.3390/ijms242015112

**Published:** 2023-10-12

**Authors:** Debora Cutuli, Laura Petrosini, Francesca Gelfo

**Affiliations:** 1 IRCCS Fondazione Santa Lucia, Via Ardeatina 306, 00179 Rome, Italy; 2 Department of Psychology, Sapienza University of Rome, Via dei Marsi 78, 00185 Rome, Italy; 3 Department of Human Sciences, Guglielmo Marconi University, Via Plinio 44, 00193 Rome, Italy

## 1. Introduction

A substance capable of inducing a consistent pattern of neural dysfunction in the chemistry or structure of the nervous system may be defined as neurotoxic. Even brief exposures to such substances can result in severe functional deficits, including abnormalities in relevant biochemical parameters, EEG findings, psychological and behavioral testing, neurological examinations, and axonopathy and cell death. Furthermore, neurotoxins produce various effects in development and aging.

This Special Issue, titled “Advance in Neurotoxicity Research from Development to Aging”, presents new insights on the lifetime effects of common neurotoxic agents on brain functioning and neural organization. In fact, despite a growing body of preclinical studies demonstrating neurotoxic effects in both developing and aging brains, the impact of neurotoxic agents on the human brain remains to be clarified. In particular, elderly subjects are at increased risk of adverse reactions given the presence of concomitant age-related physiological alterations that may affect pharmacokinetic processes.

This Special Issue includes a total of eight papers addressing the effects of the exposure to different neurotoxic agents in both developmental and/or aging stages. Namely, advances on the specific aspects of the neurotoxicity of metals (such as zinc, silver, and iron [1,2,3]), inhalational anesthetics (such as sevoflurane [4]), bacterial endotoxins (such as Lipopolysaccharide (LPS) [5,6,7]), as well as Amyloid β (Aβ)-induced cytotoxicity [8] during different stages of development (from gestational and postnatal periods to adulthood and old age) are described in the present Special Issue and will be discussed as follows:

## 2. Metal Neurotoxicity

Metals are commonly found in the environment. They may be implicated in the development of neurological symptoms and neurodegenerative illness, such as Alzheimer’s disease (AD), Parkinson’s disease (PD), Huntington’s disease, or amyotrophic lateral sclerosis [9]. Depending on the method and duration of exposure, some metals may be the sole cause of a pathological condition, while others may contribute to cause or accelerate a neurological disorder [10].

The present Special Issue highlights the neurotoxic effects of metals such as zinc, silver, and iron in the development of neurological disorders across the lifespan.

Zinc is an essential metal assimilated within the body by oral ingestion, skin exposure, and inhalation [11]. It supports healthy growth and development and plays a role in enhancing immune function. Its total amount in the adult human body is 2–3 g, being mainly stored in the muscle and bone [12]. Zinc may cause pathological effects when in excess or deficiency [13]. Neurotoxicity induced by zinc excess has been shown to be involved in neuronal damage and death associated with traumatic brain injury, stroke, seizures, and neurodegenerative diseases such as AD [14,15]. Zinc deficiency can result in lethargy, neurosensory pathology, neuropsychiatric disorders, and reduction in nerve conduction [16,17].

In the central nervous system (CNS), zinc ions (Zn^2+^) inhibit γ-aminobutyric acid type A (GABAA) receptors, pivotal mediators of inhibitory synaptic neurotransmission [18]. The inhibitory potency of zinc ions is severely affected by the subunit composition of GABAA receptors and differs during developmental stages [19]. Extra- and intra-cellular zinc affects the GABAA receptor-mediated anion transport and ATPase activity. Interestingly, it has been found that GABAA receptor-coupled ATPase is involved in seizures [20].

**Menzikov and colleagues [3]** propose a mechanistic and molecular basis for the inhibition of GABAA receptors and ATPase function by zinc during brain maturation by studying neonatal and adult rats. Namely, the Authors have demonstrated that in the rat enzymes within the scope of GABAA receptor performance, such as Cl-ATPase and then as Cl-, HCO_3_-ATPase, form during the first week of postnatal development. The Cl-ATPase form belongs to the β1 subunit, while the β3 subunit preferably possesses the Cl-, HCO_3_-ATPase activity. Furthermore, the potency of Zn^2+^ in inhibiting GABAA receptor-coupled ATPase activity is linked to the intracellular binding with cysteine. These data identifying the molecular mechanisms of the interactions between Zn^2+^ and the ATP-hydrolysis site within the receptor molecule may have clinical implications for the therapy of brain disorders (e.g., epilepsy) and could help to develop novel drug design.

Metal nanoparticles ranging from 10 to 100 nm in size have attracted much attention for their physicochemical properties [21]. In particular, silver nanoparticles (AgNPs) have high thermal and electrical conductivity as well as unique medicinal properties as fungicidal and bactericidal agents [22]. For these reasons, AgNPs are exploited in different consumer products, like water filters, clothing, cosmetics, sprays, detergents, cell phones, laptop keyboards, and toys. It should be noted that physiologically silver is present in trace amounts in the adult human body (70–90 µg), and over 99% is readily eliminated from the body by the liver and kidneys [23]. The extensive use of AgNPs has increased human exposure to them by inhalation, dermal contact, and ingestion [24]. Several reports have indicated that AgNPs induce neurotoxic effects [25,26,27,28] since nanosilver can cross the blood–brain barrier (BBB), accumulate in the brain, increase oxidative stress and inflammation, and even promote the formation of Aβ plaques and neuronal death, thus resulting in a risk factor for neurodegeneration and concurring to adverse effects on cognition and social abilities [29,30,31,32].

In line with the raising concerns about the impact of AgNPs on human health and aim to better understand the mechanisms underlying the neurotoxicity of nanosilver, **Dziendzikowska and colleagues [1]** analyzed the effects of the chronic (28 days) oral exposure to AgNPs with different types of coating used for their stabilization (citrate; bovine serum albumin, BSA; polyethylene glycol) on the antioxidant parameters, oxidative stress, and neurosteroid metabolism in the hippocampus of adult rats. Interestingly, the Authors demonstrated that AgNPs disrupted the antioxidant system and induced oxidative stress in a coating-dependent manner in the hippocampus. Furthermore, AgNPs caused changes in the hippocampal concentrations of steroids (e.g., decreased progesterone, 17α-progesterone, and testosterone levels) and downregulated the expression of genes involved in antioxidant defense and steroid synthesis and metabolism. Notably, the disruption of mitochondrial functions induced by AgNPs may modulate steroidogenesis, which is one of the key functions of mitochondria. AgNPs-treated rats also displayed deficits of spatial memory, mainly related to the integrity of hippocampal networks [33].

Overall biochemical and behavioral alterations were predominantly caused by BSA-coated AgNPs. This can be linked to the reduced stability provided by the coating material in the biological fluids, since BSA is prone to digestion by the proteolytic enzymes in the gastrointestinal tract. The toxic effects of BSA-coated AgNPs may be attributed to the release and accumulation of silver ions (Ag+) in the hippocampus that are responsible for mitochondrial dysfunction, and the study by Dziendzikowska and colleagues [1] indicates that neurotoxicity could potentially be minimized by using more appropriate coating.

Even the alteration of homeostasis of another metal, the iron, may induce neurotoxicity, shifting from physiological pathways to pathological situations. Iron is the most abundant essential metal in the adult human body, reaching an amount of 4–5 g and being present mostly in red blood cell hemoglobin (65%) or in the liver in the form of ferritin (30–35%) [34]. Neurodegeneration with brain iron accumulation (NBIA) is a group of inherited neurologic disorders characterized by iron deposition in the globus pallidus and the substantia nigra and resulting in progressive movement disorders (dystonia, spasticity, chorea, parkinsonism), psychomotor retardation, ocular abnormalities, neuropsychiatric disorders, and cognitive decline [35]. Iron accumulation is also a recurring pathological phenomenon in various sporadic age-related neurological diseases such as PD and AD [36]. Remarkably, it has been observed that brain aging processes per se are responsible for increased iron levels, which can lead to oxidative cellular damage and reactive gliosis and thus contribute to cognitive deficits [37,38]. About that, in their recent review paper, **Ficiarà and colleagues [2]** collected the available data on the brain iron concentrations throughout the lifespan, especially during aging. Age-related iron accumulation found in the brain, and particularly in hippocampus and basal ganglia, seems to be correlated with decreased memory performances [37]. Recent evidence suggests that the propensity of brain cells to accumulate excessive iron as a function of aging largely depends on their anatomical location and vasculature and on the differential deterioration of the BBB structure [2]. For example, quantitative MRI methods (e.g., quantitative susceptibility mapping, QSM) and post-mortem studies indicated an increase in iron content in deep grey matter regions (such as the basal ganglia) during aging [39,40,41]. In addition, Ficiarà and colleagues [2] identify genetic factors (presence of apolipoprotein E 4, APOE-ε4), ferroptosis (i.e., iron-dependent programmed cell death), and microbleeds and intracerebral hemorrhage as concurring factors able to increase the brain iron content until it reaches values predicting neurodegeneration. Namely, it has been reported that cerebral microbleeds are more prevalent in AD patients than in the general population [42] and senile plaques are sites of microhemorrhages [43]. Thereby, an early and accurate detection of iron content in the brain, based on quantitative models of physiological and pathological iron exchange, can allow the use of iron as a biomarker in prodromal stages of age-related neurodegenerative disorders and to intervene therapeutically more promptly to counteract inflammation and oxidative stress.

## 3. Anesthetic Neurotoxicity

General anesthetics act on several receptors in the CNS (such as GABA receptors), and their effects are generally reversible. However, in some patient populations there is an increased risk of neurotoxic side effects, including memory and cognitive impairments. In fact, perioperative neurocognitive disorders are repeatedly observed in postoperative patients [44].

Although sevoflurane is a volatile general anesthetic agent commonly used in clinical practice, its effects in postoperative subjects are still debated since in animal studies it has been reported that it could exert either neuroprotective (e.g., in cerebral ischemia, hemorrhagic shock, and LPS-induced neuroinflammation models) or neurotoxic (e.g., in pregnancy and type 2 diabetes models) effects.

It has been hypothesized that pre-existing mild cognitive impairment accompanied by accumulated neuropathology (such as tau and Aβ aggregates) may be a risk factor for sevoflurane neurotoxicity. To test this, **Huang and colleagues [4]** have investigated the effects of sevoflurane in the 3 x Tg mouse model of AD. It is a widely used model in which mice express three mutations associated with familial AD (APP Swedish, MAPT P301L, and PSEN1 M146V) and display both Aβ plaques and tau tangles [45,46]. The prolonged inhalation (2 h) of sevoflurane exacerbated cognitive deterioration and neuronal dysfunction (including disrupted synaptic glutamatergic transmission and increased neuronal apoptosis) in 3 x Tg mice but not in wild-type mice, thus demonstrating that sevoflurane neurotoxic effects depend on the neuropathology background [4]. This study helps to explain the previous findings showing that aging is a risk factor for postoperative cognitive dysfunction since different neuropathological hallmarks, including tau aggregation, deposition of amyloid plaques, and neurofibrillary tangles, are usually found with increasing age. Furthermore, the findings by Huang and colleagues [4] underline the importance of preserving glutamate homeostasis and focusing on the preoperative assessment to evaluate potential risk factors. This is so alternative anesthetics may be used for patients with AD neuropathological signs. In this context, propofol is a general intravenous anesthetic drug which has proven to have a minimal impact on behavior and pathological changes in AD mouse models [47,48].

## 4. Lipopolysaccharide Neurotoxicity

Lipopolysaccharide (LPS) is a glycolipid derived from the microbiome and is an extremely potent neurotoxin that causes inflammation. In humans, the main sources of LPS are Gram-negative bacilli found in the gastrointestinal tract, such as Bacteroides fragilis and Escherichia coli [49]. Evidence is available on the consistent detection of LPS in the brains of elderly humans, and its presence has been found to increase around and within neurons affected by AD [5]. LPS and other toxins generated by the microbiome can cross the barriers of the gastrointestinal tract and the BBB, particularly in aging individuals and those with vascular disorders like leaky gut syndrome [50]. The literature also suggests that LPS enhances the activity of the pro-inflammatory transcription factor complex NF-kB (p50/p65) and subsequently upregulates a group of microRNAs (including miRNA-30b, miRNA-34a, miRNA-146a, and miRNA-155). These upregulated microRNAs, in turn, downregulate a family of messenger RNAs (mRNAs) associated with neurodegeneration, including the mRNA that encodes the neuron-specific neurofilament light (NF-L) chain protein [51]. While NF-L has been found to be increased in peripheral biofluids in AD and other inflammatory neurodegenerative diseases, it is significantly decreased within neocortical neurons. This may explain the atrophy of neurons, loss of axonal size, changes in neuronal cell shape, modifications in synaptic structure, and deficits in neuronal signaling capacity [5]. When experimentally administered, LPS induces non-specific and acute reactions that trigger systemic inflammation, which has been implicated in various common diseases. These states include maternal immune activation (MIA) and the pathogenesis of cardiovascular diseases, chronic kidney diseases, autoimmune diseases, cancer, depression, and neurodegenerative diseases [52,53]. LPS binds to the cluster of differentiation 14 (CD14)/Toll-like receptor 4 (TLR4)/myeloid differentiation factor 2 (MD2) receptor complex present in monocytes, dendritic cells, macrophages, and B cells within the host system. The subsequent response is cell-dependent. The activation of TLR4 leads to the production of various inflammatory mediators such as tumor necrosis factor (TNF) and interleukins (IL-1, IL-6, IL-8, IL-18), which stimulate the release of prostaglandins and leukotrienes, ultimately resulting in inflammation and septic shock, the key features of endotoxemia [54]. Additionally, TLR4 activation is essential for adaptive immune response by promoting the production of co-stimulatory molecules. Inflammatory mediators play a crucial role in driving behavioral changes, such as sickness behavior, which is the immediate consequence of cytokine elevation [55]. TLR4 activation also causes the release of histamine, leading to vasodilation, and activates clotting factors associated with issues like thrombosis, acute disseminated intravascular coagulation, hemorrhage, and septic shock [56].

**Zhao and colleagues [5]** reviewed the literature to reveal the most current findings on LPS neurotoxicity. They investigated how such pro-inflammatory glycolipids contribute to the biological mechanisms of progressive, age-related, and ultimately lethal neurodegenerative disorders. The Authors report the recent discoveries on the gut-microbiota-derived LPS–NF-kB–miRNA-30b–NF-L pathological signaling network. They highlight that a direct pathological connection exists between the LPS of gastrointestinal tract microbes and the inflammatory neuropathology, disorders of cytoskeleton, and disrupted synaptic signaling of the AD and/or stressed human brain cells in primary culture. Moreover, they describe such a link as the first example of a microbiome-derived neurotoxic glycolipid having significant detrimental miRNA-mediated actions on the expression of NF-L, a filamentous protein known to be important in the maintenance of neuronal and synaptic homeostasis.

However, in relation to LPS neurotoxicity, two papers proposed in the present Special Issue describe the protective effects of two different kinds of non-pharmacological interventions, such as Environmental Enrichment (EE) [7] and dietary supplementation [6].

**Landolfo and colleagues [7]** focused their review on the beneficial effects of EE, defined as a combination of enhanced stimulations involving cognitive, social, and physical factors [57,58]. EE results in a wide range of brain changes, such as increased synaptogenesis, neurogenesis, glycogenesis, angiogenesis, and modulations of neurotrophic factors and neuroinflammation, inflammation, immune senescence, and DNA epigenetic modification, with the resulting modulation of cognition and behavior [59,60]. The aim of the review was to describe the effects of the exposure to EE paradigms in counteracting LPS-induced neuroinflammation throughout the lifespan. On the whole, the Authors report that the exposure to EE is able to exert sex- and age-dependent neuroprotective and therapeutic effects on LPS-induced neurotoxicity. Such effects are present both in rodents with generic LPS-induced neuroinflammation and in pathological models where LPS injection is used to develop a specific condition. Evidence is available throughout life, namely, on LPS administration in the prenatal period (to the mothers), in early age, and throughout adult life until old age. Although the Authors highlight that further experiments will be needed to fully understand the mechanisms underlying EE’s beneficial actions in case of neurotoxic exposure to LPS and to study which aspects of EE (exercise, socialization, cognitive stimulation) represent the critical ingredients to enhance brain plasticity in the case of neurotoxic exposure to LPS, the reported findings propose that an efficient and tuned use of EE protocols may allow the management of neuroinflammatory damage even in clinical settings.

**Decandia and colleagues [6]** focused their literature review on the beneficial effects of dietary supplementation. It is well known that diet modulates the immune system and that different nutrients and bioactive components can influence neuroinflammation [61]. Polyphenols, unsaturated fats, and vitamins A, C, and E inhibit oxidative stress and neuroinflammation, while saturated fats promote neuroinflammation, particularly in the hypothalamus [62,63]. In particular, the review aimed to describe the potential of specific dietary compounds to counteract cognitive decline, neuroinflammatory, and/or oxidative stress correlates in AD-like animal models of LPS-induced neuroinflammation. The compounds reviewed include curcumin, krill oil, chicoric acid, plasmalogens, lycopene, tryptophan-related dipeptides, hesperetin, and selenium peptides. Despite the heterogeneity of these compounds, the paper reveals a strong consensus on their counteracting action on LPS-induced cognitive deficits and neuroinflammatory responses by modulating cell signaling processes, such as the NF-B pathway, and supports that dietary interventions may represent an important resource to oppose AD, by modulating neuroprotection and immune regulation.

## 5. Amyloid β-Induced Cytotoxicity

Finally, the focus of the article by **Wang and colleagues [8]** was the growing evidence suggesting that one of the underlying causes of brain function decline is the excessive production and improper folding of the Aβ peptide, which leads to the formation of toxic aggregates known as oligomers and fibrils [64]. Suppressing the aggregation of Aβ could be a potential treatment option in order to reduce the harmful effects on the brain synaptic connections. In recent years, considerable efforts have been made to develop various molecules that specifically target and inhibit Aβ aggregation, thereby opening a promising avenue for the early intervention and prevention of AD [65]. However, most of these inhibitors have shown limited effectiveness as they are easily degraded in the blood and have poor ability to cross the BBB [66].

In their study, Wang and colleagues [8] describe a new approach to treating AD, which involves the use of a combination of photothermal and photo-oxygenation techniques. They propose a nano-platform composed of the brain-targeting peptide RVG, the 2D porphyrinic PCN-222 metal–organic framework, and indocyanine green (PCN-222@ICG@RVG). This combination treatment shows the improved photoinhibition of Aβ aggregation in AD. The Authors report that the photothermal and photo-oxygenation treatment, when based on PCN@ICG, significantly increased the inhibition of Aβ-42 aggregation. Furthermore, this treatment resulted in lower neurotoxicity when exposed to near-infrared (NIR) irradiation at 808 nm compared to single-modality photo treatment in both cell-free and in vitro experiments. The increase in local photothermal heat destabilizes Aβ aggregates, keeping them in their monomeric state and facilitating the generation of oxidized Aβ monomers with a reduced aggregation capability. Additionally, the Authors report that the inclusion of the brain-targeting peptide RVG in the PCN-222@ICG@RVG nanoprobe facilitates its permeability through the human BBB in a human brain-on-a-chip model. Ex vivo studies have also demonstrated that NIR activation of PCN-222@ICG@RVG effectively disassembles Aβ plaques. Based on these findings, the authors propose that the synergistic combination of photothermal treatment with photo-oxygenation has the potential to enhance the inhibition of Aβ aggregation and may contribute to future NIR-based combinational phototherapy for AD.

## 6. Conclusions

In summary, the present Special Issue includes four research articles—providing in-depth insights into the molecular mechanisms underlying neurotoxicity induced by the exposure to specific metals (i.e., zinc and silver), anesthetics (i.e., sevoflurane), as well as Aβ-induced cytotoxicity—and four comprehensive reviews—examining the broader mechanisms underlying neurotoxicity induced by the exposure to iron and endotoxins, such as LPS. The described mechanisms are investigated throughout the different stages of development. Some of the papers provide advancements on therapeutic strategies to cope with neurotoxicity effects, mainly proposing non-pharmacological interventions (such as complex environmental stimulations, dietary supplementation, and near-infrared phototherapy).
